# Raman scattering study on Sb spray InAs/GaAs quantum dot nanostructure systems

**DOI:** 10.1186/s11671-015-0908-1

**Published:** 2015-04-29

**Authors:** Liping Dai, Stephen P Bremner, Shenwei Tan, Shuya Wang, Guojun Zhang, Zongwen Liu

**Affiliations:** State Key Laboratory of Electronic Thin Films and Integrated Devices, University of Electronic Science and Technology of China, No 4, Section 2, Jianshe North Road, Chengdu, 610054 China; Australian Centre for Microscopy and Microanalysis, The University of Sydney, Chemical Engineering Building, Sydney, 2006 Australia; School of Photovoltaic and Renewable Energy Engineering, University of New South Wales, Sydney, 2052 Australia

**Keywords:** InAs, Quantum dots, Raman spectra, Sb spray

## Abstract

The effect of Sb spray time on the structure of InAs/GaAs quantum dot (QD) systems with Sb spray prior to the capping of a GaAs layer was determined by a Raman scattering study. The Raman spectra of the InAs/GaAs system show two phonon signal bands related to interface (IF) defects, located at the low-energy side of InAs QDs and GaAs cap layer main phonon peaks, respectively. The intensity ratio of the IF defect relative phonon signal to its corresponding main peak shows a significant decrease with the Sb spray time increasing from 0 to 15 s, but increases for spray times larger than 15 s. In addition, the InAs QD phonon peaks appear to be resolved with improved symmetry for 15 s of spray time. Finally, the GaAs transverse optical (TO) phonon peak is seen to vary with Sb spray time, both in terms of the intensity and the peak position, in a similar manner to the other results. Taken together, these results suggest the InAs/GaAs QDs with a 15-s Sb spray lead to a GaAs capping layer with less strain at the IF with the QDs and a lower density of crystalline defects.

**PACS:** 81.05.Ea; 81.07.-b; 81.07.Ta

## Background

Semiconductor quantum dots (QDs) and other nanomaterials have a great potential for applications in a wide variety of novel devices [1-4]. In recent years, the III-V QDs, especially InAs/GaAs, have been drawing great interest due to their promise in wide applications [4,5]. However, their emission wavelength of around 1,200 nm is not interesting for applications in telecommunication devices which work most effectively at wavelengths of around 1.550 μm [6]. A significant effort has been made in the last few years to extend the emission wavelength of InAs/GaAs QDs to the 1.55-μm telecommunication band by a number of different approaches [6-10], such as growing larger QDs by deposition of more QD material, by controlling the epitaxial growth conditions, or by growing on high-index surfaces. In addition, another important method is by embedding other compositions in an InGaAs matrix [11-13], which results in the increased aspect ratio of the QDs and hence reduced strain inside the QDs, causing a redshift in the emission. The effect of an alternative matrix layer has also been investigated for InAs/GaAs QDs capped with other materials, such as InGaAsN [14] and Graphene [15]. The inclusion of antimony (Sb) is an interesting approach that can be used for InAs/GaAs QD structure tuning. A strong emission wavelength redshift has been observed when using GaAsSb instead of GaAs capping layers for InAs/GaAs QDs [16,17]. Several reports about the structure of these QDs have demonstrated significantly different properties from those of GaAs-capped QDs by means of transmission electron microscopes (TEM), cross-sectional scanning tunneling microscopy (X-STM), and atomic force microscopy (AFM) [18-21].

The effects of a Sb spray immediately prior to GaAs capping on the InAs/GaAs QD structure have had some reports, but there are still many aspects that are not understood completely. In previous work [22], photoluminescence of InAs/GaAs QDs treated with different Sb sprays prior to capping with GaAs showed that the intensity and wavelength of emission varied with the Sb spray doses. These results may be related to the structure variation with Sb spray treatment. In this work, we have investigated the basic structure of InAs/GaAs QD systems by TEM and have used a simple, non-invasive method of Raman scattering to characterize the evolution in the microstructure of the InAs/GaAs QD systems with different Sb spray times. The intensity ratio of InAs and GaAs IF to its main peak transverse optical (TO) phonon as a measure of the quality of the crystal structure of InAs QDs and GaAs IF as well as the corresponding GaAs TO peak intensity and shift with the Sb spray time are compared and explained. And a statistical insight on the QD system structural quality was also obtained.

## Methods

Four samples studied were grown by molecular beam epitaxy in an Applied Epi Gen III system (Veeco, Plainview, NY, USA) on (100) GaAs substrates. One sample with InAs/GaAs QDs capped by GaAs with non-Sb spray was named sample 1; the other three samples with InAs/GaAs QDs sprayed by Sb flux 7.5, 15, and 22.5 s prior to a GaAs capping layer were named samples 2, 3, and 4, respectively. Gallium and indium fluxes were supplied by conventional thermal sources, and As and Sb fluxes were provided by valved cracker sources. The growth rates determined by monitoring the RHEED oscillations were for GaAs and InAs 0.4 and 0.035 monolayer/s, respectively, and the measured beam equivalent pressure for Sb was 9.7 × 10^−8^ Torr. The As overpressure for all the GaAs and InAs growth steps was 2 × 10^−6^ Torr. Approximately 2.0 monolayers of InAs were deposited on the substrates. And different growth processes were then employed for the four samples. Sample 1 had a 30-s rest under As flow, and samples 2, 3, and 4 were exposed to the Sb flow 7.5, 15, and 22.5 s and then had 22.5-, 15-, and 7.5-s As soak, respectively. This means that each of the samples gets a 30-s total group V soak. At the end of each group’s spray regime, a 30-nm GaAs cap layer was grown immediately.

The morphology and structural characteristics of InAs/GaAs QDs with non-Sb and 7.5-s spray were investigated by cross-sectional TEM with a JEOL-JEM-3000 F microscope (JEOL Ltd., Akishima-shi, Japan) operated at 300 kV. Raman scattering measurements were performed in air at room temperature in backscattering geometry with a Renishaw InVia Reflex System (Renishaw, Wotton-under-Edge, UK) using the 514-nm line of a CW laser as excitation with a spot diameter of about 1 μm. The laser output power was fixed at 10 mW so as to avoid excess heating of the samples and was focused onto (100) surfaces by using a cylindrical lens. Taking into account the selection rules of RS in backscattering geometry along a (100) surface, LO phonons should be only allowed in parallel polarization, while TO modes should be forbidden.

## Results and discussion

**Figure 1:**
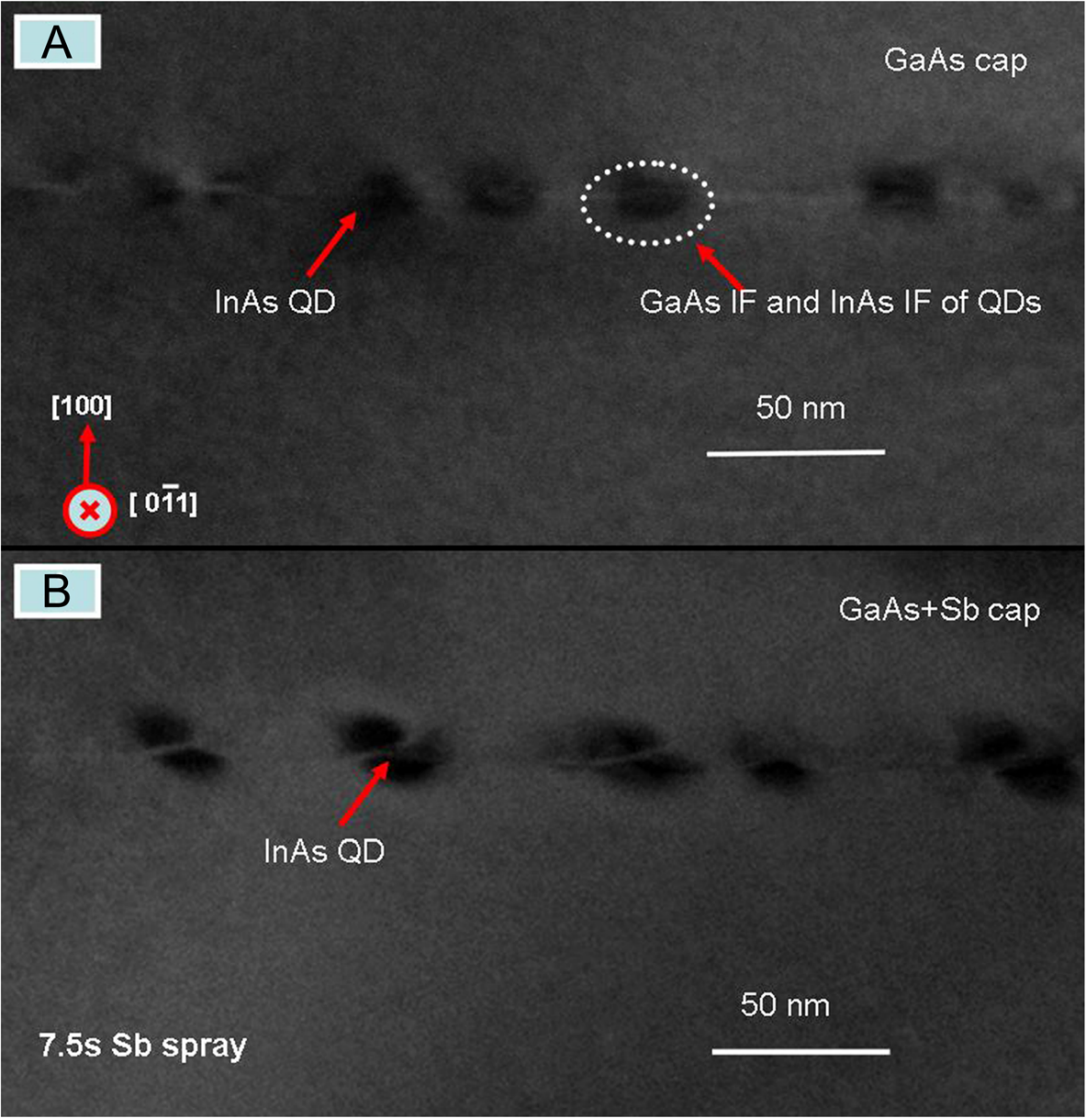
The cross-sectional TEM images of InAs/GaAs QD systems. **(A)** Sample 1 with non-Sb spray. **(B)** Sample 2 with the 7.5-s Sb spray.

Low-magnification [1-10] cross-sectional TEM images of samples 1 and 2 are shown in Figure 1A, B for a general impression of the InAs/GaAs QD system nanostructure. One layer of buried InAs/GaAs QDs is observed according to the dark contrast caused by the strain field around the capped QDs [23]. Figure 1A presents a typical InAs QD shape of the lens [24], with a height of 5 ± 1 nm, a base width of 12 ± 2 nm, and an interspacing of QDs in a range of 15 to 25 nm. As shown in Figure 1B, the Sb spray InAs QDs have a truncated pyramid-like shape with a wider base and higher height and show an obvious asymmetry in dark contrast for the stress variation [24]. In order to have an insight of the effects, Raman scattering, as a simple tool, was used to research the variation of the nanostructure systems by the Sb spray treatment with different times.

**Figure 2:**
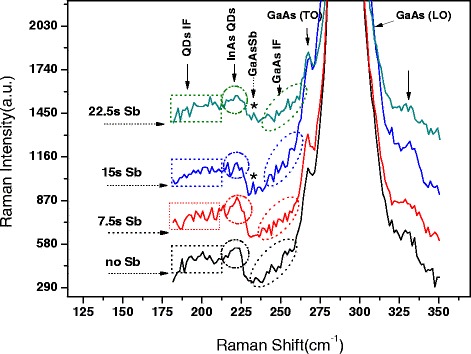
The Raman spectrum of InAs/GaAs QD systems of the four samples (treated with no Sb, 7.5 s Sb, 15 s Sb, and 22.5 s Sb, respectively).

Figure 2 displays the Raman spectrum of InAs/GaAs QD systems of the four samples which were treated with 0, 7.5, 15, and 22.5 s Sb, respectively. In our experiment mode, the peaks of interest are found in the 200 to 300 cm^−1^ range, a very strong peak at 293 cm^−1^, and a weak peak at 273 cm^−1^, which are attributed to the LO and TO modes of GaAs, respectively [15,25]. As mentioned above, only the LO mode is allowed for a (100)-oriented material and the TO mode is, strictly speaking, forbidden. However, a small peak due to the TO mode is also observed; it is probably due to a slight substrate misorientation or imperfection. Another possible cause may be a small experimental deviation from the backscattering. This phenomenon has also been observed by other semiconductors with (100) orientation [15]. At the low-energy side of the GaAs TO, a satellite peak band marked by an oval centered at 250 cm^−1^ can be observed, which can be attributed to the GaAs relative QD interface (IF) phonon signal (named GaAs IF for short). This IF mode is associated with the InAs QD edges [26]; therefore, this weak band does not appear in the bulk GaAs materials [27,28]. There, the small shake peaks have different phonon signal centers, which are considered to originate from different degrees of In/Ga intermixing on the QD IF.

From the spectra, we can see the phonon signal band of GaAs IF has a high-energy side shift and shows an obvious variation in the shake peak numbers and intensity with increasing Sb spray time. Especially, the InAs/GaAs QD systems with the 15-s Sb spray clearly display a decrease in shake peak numbers and a strengthening in the Raman intensity of GaAs IF signal. The results are assumed to originate from the Sb intermixing in the GaAs matrix, with varied compositions, stresses, and defects being manifested in the GaAs IF signal. Due to the lattice mismatch between InAs and GaAs, during the epitaxial growth, the strain will accumulate and result in the formation of lattice deformations or dislocations. These defects may be located in the InAs IF, inside the InAs QDs, or in the GaAs IF. Therefore, the up-shift, intensity, and peak number variations of the GaAs IF Raman signal can be explained in the following manner: during the Sb spray treatment, larger lattice constant GaAsSb is formed which results in reducing the QD/capping layer IF lattice mismatch. With the Sb spray time increased to 15 and 22.5 s, we can observe the obvious phonon signal for the Ga-Sb bonds located at the frequency of 230 cm^−1^ [29,30]. The presence of GaAsSb changes the stress distribution at the QD/cap IF, leading to a shift in the GaAs IF phonon line to higher energy. At a certain Sb spray treatment, the symmetry of the strain will achieve an optimum value, and the defects and dislocations will be reduced to a large degree. Furthermore, the uniformity of the GaAs IF In/Ga intermixing is enhanced. Therefore, the phonon signal peak of GaAs IF will be strengthened, as shown in the spectra; the 15-s Sb spray treatment is just the case.

The above results are also supported by the InAs QD phonon line signal. On the low-energy side, a main phonon peak marked by a circle centered at the frequency of 220 cm^−1^ and a shake band marked by a square centered at 200 cm^−1^ are observed [15]. These are the peaks of InAs QD and InAs QD IF phonon signals, respectively. Due to the nanoscale size of the InAs QDs, the phonon frequency is less than the corresponding bulk material [31], in keeping with previous results for nanostructures such as quantum dots, wires, and nanowires, where a downward frequency shift and line width broadening of the TO and LO phonon modes are observed. This change of phonon mode frequency and line width is considered to be the relaxation of the *q* = 0 selection rule in the Raman scattering due to quantum confinement [32,33]. As can be seen from the spectra, the InAs QD phonon peak of sample 1 (no Sb spray) shows an obvious asymmetrical shape, which can be attributed to the strain caused by lattice mismatch and defects at the IF of QDs [31], and the stress was relived after Sb spray, just as seen in Figure 1B. QDs show the asymmetrical dark contrast most likely related to the formation of graded GaAsSb immediately adjacent to the InAs QDs that provides strain relief for the dot/capping layer lattice mismatch. Therefore, the phonon peak asymmetry is improved with increasing Sb spray time to 7.5 and 15 s. The LO and TO modes become increasingly resolved, with the InAs TO phonon signal as the main peak, and the LO signal giving a high-energy-side shoulder. The sample with the 15-s Sb spray shows a clear shoulder with the main phonon signal, demonstrating there is less strain occurring at the QD/cap IF in this sample, since phonon signals of unstrained materials can be sufficiently separated in energy, meaning the Raman phonons should be well resolved [31]. With the Sb spray time increased to 22.5 s, however, the InAs QD phonon peaks present broaden and decrease in intensity, with the peaks becoming less resolved. In addition, the corresponding QD IF peaks show a variation in intensity with the increase of Sb spray time, with a decrease in intensity for increasing Sb spray time from 0 to 15 s but an increase when the Sb spray time is increased to 22.5 s. The QD IF peaks show the weakest intensity for the sample with the 15-s Sb spray treatment, demonstrating less strain and associated defects occurred under this treatment condition. It should be noted that the signal peaks banded as a shoulder at the high-frequency side of the GaAs LO phonon peak remain not identified; it may be the second-order phonon peak of the IF relative signal of Ga/Sb/In/As.

**Figure 3:**
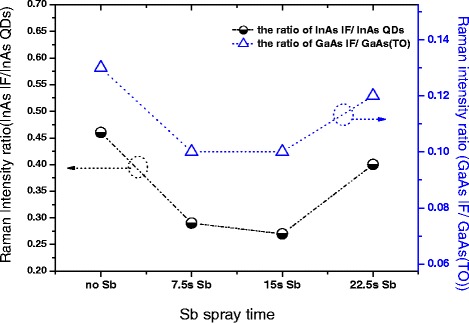
The Raman intensity ratio *I*_InAs IF_/*I*_InAs QDs_ and *I*_GaAs IF_/*I*_GaAs TO_ versus samples with different Sb spray times.

In order to further strengthen the results, the Raman intensity ratio *I*_InAs IF_/*I*_InAs QDs_ and *I*_GaAs IF_/*I*_GaAs TO_ versus samples with different Sb spray times was calculated with the results shown in Figure 3. Here, the *I*_InAs IF_ and *I*_GaAs IF_, related to the IF defects and dislocations of the phonon signal, are the average intensity of IF shake peaks, and the *I*_InAs QDs_ refers to the intensity of the InAs QD TO main phonon peak. Thus, *I*_InAs QDs_ and *I*_GaAs TO_ originate from the internal lattice material [34,35], while the *I*_IF_/*I*_TO_ ratio is associated with surface or IF defects, allowing qualitative comparison of the surface or IF defects in different samples. From the spectra, we can see the two ratios show a similar variation trend with Sb spray time, in the range of 0 to 15 s; the ratios decrease with time increasing and increase with the time up to 22.5 s, but the ratios are still less than those of the 0-s Sb spray of sample 1. The two lowest values both appear in sample 3 (15-s Sb spray treatment), so, combined with the above discussion, the 15-s Sb spray is considered to be an optimal parameter for decreasing the defects of the InAs/GaAs QD nanostructure systems.

**Figure 4:**
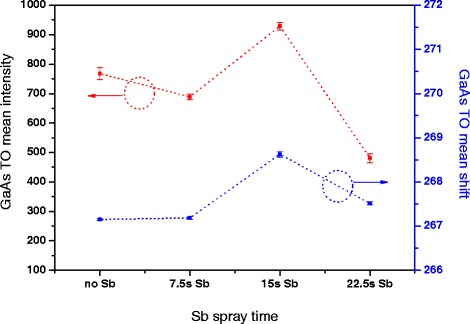
GaAs TO intensity and Raman shift variation with Sb spray time. The error bars are standard deviations and standard errors of the mean.

In order to determine the reliability of the above results, we repeated the Raman experiment several times and show the results using the error bars of the intensity and Raman shift of GaAs TO peak varying with Sb spray time, as in Figure 4. The two lines show a similar trend, with the mean of Raman intensity and Raman shift both obtaining a maximum for the 15-s Sb spray. This demonstrates the release in stress and concomitant decrease in defects in the GaAs lattice structure for this treatment, a result in agreement with the above discussions. Although the Raman frequency shift variation of the GaAs TO signal is found to not exceed 2.0 cm^−1^, far less than that of corresponding GaAs IF shift, the results are still reliable. The presence of the TO signal, due the GaAs bulk crystal structure, instead of surfaces and IFs, and the standard deviations being less than 0.85, with standard errors less than 0.37, results in a tight point distribution around the mean, indicating a high reliability of the experimental results.

## Conclusions

Raman spectra of InAs/GaAs QD samples with different Sb spray times prior to QD capping have been presented. Raman signals of GaAs and the InAs QDs are observed, along with GaAs IF and InAs QD IF signal bands at the low-energy side of the main peaks. It was found that the two ratios of *I*_InAs IF_/*I*_InAs QDs_ and *I*_GaAs IF_/*I*_GaAs TO_, which indicate the relative defect densities of the samples, are at a minimum for the 15-s Sb spray treatment. Additionally, for the 15-s Sb spray sample, the InAs QD phonon signal peaks show maximum symmetry and resolvability compared to that of other samples. The small error bars of Raman shift and intensity of GaAs TO demonstrate a high reliability of the experimental results, giving greater confidence in the above observations. The results, taken together, indicate the 15-s Sb spray treatment is optimal for decreasing the defects and releasing the stress of InAs/GaAs QD nanostructure systems. This is attributed to the formation of GaAsSb at the QD/cap IF, resulting in reducing the crystal mismatch with InAs. This study provides a simple, non-invasive method of Raman spectra to investigate the effects of different Sb spray times on the structure of InAs/GaAs QD nanostructure systems.
